# On the Reducible Character of Haldane-Radić Enzyme Kinetics to Conventional and Logistic Michaelis-Menten Models 

**DOI:** 10.3390/molecules16043128

**Published:** 2011-04-13

**Authors:** Mihai V. Putz

**Affiliations:** Laboratory of Computational and Structural Physical Chemistry, Chemistry Department, West University of Timişoara, Pestalozzi Street No.16, Timisoara, RO-300115, Romania; Email: mv_putz@yahoo.com or mvputz@cbg.uvt.ro; Tel.: +40-(0)256-592-633; Fax: +40-(0)256-592-620

**Keywords:** W-Lambert equation, logistic transformation, cholinesterases, fitting curves, kinetic parameters

## Abstract

The conceptual and practical issues regarding the reduction of the Haldane-Radić enzymic mechanism, specific for cholinesterase kinetics, to the consecrated or logistically modified Michaelis-Menten kinetics, specific for some mutant enzymes, are here clarified as due to the limited initial substrate concentration, through detailed initial rate and progress curve analysis, even when other classical conditions for such equivalence are not entirely fulfilled.

## 1. Introduction

**Scheme 1:**
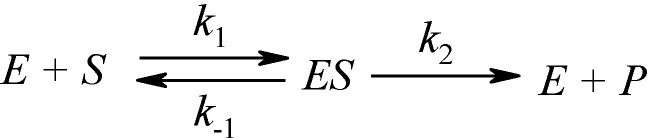
The Michaelis-Menten enzyme kinetics flowing mechanism.

Dating back now more than a century since firstly proposed by Henri in 1901 [1] the general Michaelis-Menten mechanism of enzyme kinetics (1913) [2] assumes that when an enzyme E acts upon a substrate S the complex ES is formed which in turn is converted into a product P and enzyme, according to Scheme 1 [3,4,5,6,7,8].

The model of Scheme 1 is mainly employed through its working equation [9]:

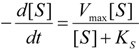
(1)
for determination of the kinetic parameters as the maximum rate of reaction:

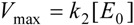
(2)
and of the Michaelis rate reaction constant:

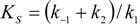
(3)
by fitting various substrate concentrations against the recorded activity (product formation); alternatively, the progress curves were used by numerically integrating Equation (1) [10] until the discovery of the W-Lambert closed form solution [11,12,13,14]:

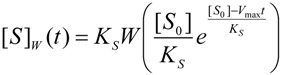
(4)
in terms of the so called omega- or Lambert *W*(*x*) function viewed as a generalization of a logarithm. It fulfills the baseline equation [15,16]:

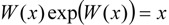
(5)
and can be used to represent real solutions for a variety of transcendental equations providing *x*>-1/*e*.

However, the role of the Michaelis-Menten enzyme kinetic mechanism in various biochemical effects was successfully employed by its adaptation for elucidation of the enzymic structure modification with the aid of chemical modifications of functional groups [17], along with the so called “forced evolution” [18], up to its recently biotechnologically recognized importance in modeling site-directed mutagenesis [19,20] or gene-shuffling techniques [21].

**Scheme 2:**
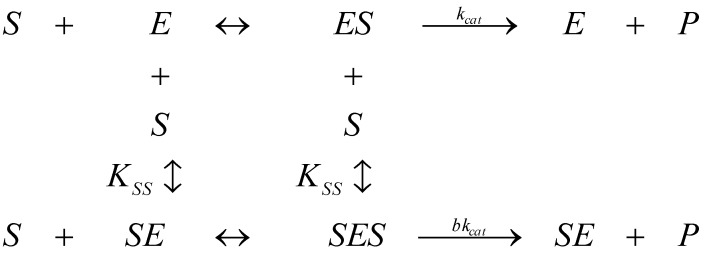
The Haldane- Radić enzyme kinetics flowing mechanism.

Nevertheless, Michaelis-Menten Scheme 1 is not a universal paradigm for enzyme modelling, especially when considering kinetics of cholinesterases from various sources. However, even for that, it was revealed, for instance, that the Glu^199^→ Asp^199^ mutation in the sequence Phe-Glu-Ser-Ala-Gly at the active center of the three-dimensional structure of *Torpedo californica* acetylcholinesterase (AChE; EC 3.1.1.7) surprisingly appears to affect similarly the binding of the peripheral and active center site ligand; as a consequence the allosteric coupling between the sites is diminished, and the substrate inhibition is no longer observed indicating the substrate inhibition constant (*K_SS_*) becomes infinitely large:

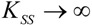
(6)
while the catalytic activity exceeds that for the wild-type enzyme for the high substrate concentration towards Michaelis-Menten kinetics [22]. Equally effects and elements of substrate activations were recorded for butyrylcholinesterases (BuChE) specific mutants such as the F_297_I, F_338_G, F_297_Y, or D_74_N of mouse AChE-BuChE enzyme chimeras [23]. Such allosteric studies introduced the conceptual need the substrate (S) may combine at two discrete sites of an enzyme forming two binary complexes ES and SE that both end up within the ternary complex SES whose hydrolysis efficiency relative to the Michaelis-Menten binary complex ES is now quantified by the catalytic parameter (*b*), see Scheme 2.

Note that in Scheme 2 it was assumed, paradigmatically, that the substrate combines equally well with enzyme and the complex ES. Therefore, Scheme 2 principally reduces to that of Michaelis-Menten in cases where there is little substrate inhibition, as provided by Equation (6) above, or when the value *b* approaches unity:

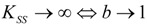
(7)

Further variants of Scheme 2 were also considered in regarding the modeling of inhibition of *Drosophila melanogaster* acetylcholinesterase active site gorge trying to furnish a putative model for the essentially not-Michaelis-Menten kinetics of cholinesterases in general, and those of insects in special, such that to combine activation and inhibition for a large range of substrate concentrations [24,25,26]. Nevertheless, all these inhibition models originate into the classical Haldane equation [27]:

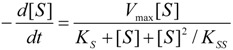
(8)
having also been used, besides enzyme kinetics, to describe biodegradation and respirometric studies involving inhibitory substrates [28,29]; yet, due to the transcendental equation furnished upon direct integration, Equation (8) has no analytical solution, unless approximate serial decomposition method is used [30], with a reliability strongly depending by time-intervals considered, while the kinetic parameters are determined based on an initial estimate followed by recursive improvements.

In this context, the present paper explores the temporal solution for the substrate traffic in the Haldane-Radić enzyme kinetics presented in Scheme 2 as it will be formulated either by closing W-Lambert analogously form of Equation (4) or even as analytical progress curves for identifying the cases its reduction to the Michaelis-Menten enzyme kinetics of Scheme 1 may be validated.

## 2. Background Theories

### 2.1. Haldane-Radić Equation

Here we derive the working kinetic equation for the enzyme model of Scheme 2. One starts with considering the specific kinetic parameters such as the maximum velocity:

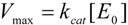
(9)
and those of equilibrium constants:

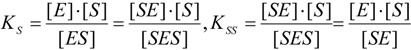
(10)

Next, by employing the global velocity expression:

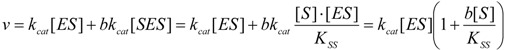
(11)
there appears the need for ES concentration knowledge; it can be nevertheless determined through the enzymic conservation equation:

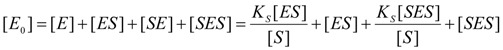


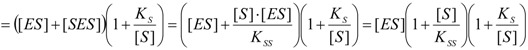
(12)
with the form:

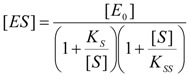
(13)

Finally by substituting Equation (13) into (11) one gets the Haldane-Radić equation for substrate excess inhibition (*b* < 1) and activation (*b* > 1):

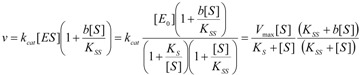
(14)
as a natural generalization for the Haldane equation (8). There is immediate the equivalence of the Haldane-Radić equation (14) with the Michaelis-Menten counterpart of Equation (1) when the equivalent conditions of (7) apply. Once learned how Haldane-Radić enzyme equation and mechanism reduces to that of Michaelis-Menten, one further likes to have the solution of the Equation (14) for its substrate temporal evolution. To this aim the preliminary benchmark Michaelis-Menten progress curve analysis will be next exposed within the recently advanced probabilistic method in enzyme kinetics [31,32,33,34]

### 2.2. Logistic Enzyme Kinetics

Within the probabilistic approach the substrate-binding equation may take the general form [31]:

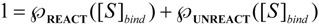
(15)

In equation (15), 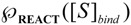 is the probability that the enzymatic reaction of Equation (1), for instance, proceeds at a certain concentration of substrate binding 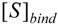 to the enzyme; it features the limits of the occurrence of products in E-S reactions:

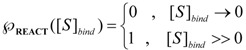
(16)

We observe that the upper branch of (16) corresponds with the case the enzymatic reaction does not proceed or when it stops because the substrate fails to bind or is entirely consumed. Instead, the lower branch of (16) describes the proceeding of the enzymatic reaction as it is related to the standard quasi-steady-states approximation (QSSA) [35,36].

On the other hand, the term accounting for the “inhibition” of the enzymatic catalysis in (15) has the reverse probabilistic range, namely:

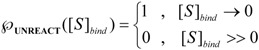
(17)

This probabilistic picture may be exemplified for the classical Michaelis-Menten Scheme 1 by recognizing that the binding substrate concentration can be treated as the instantaneous substrate concentration, *i.e.*
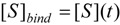
, followed by identification of the reactive term (16) as having the general form:

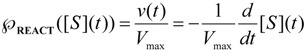
(18)
since modeling the rate of consumption of the substrate to saturation after the initial transient of the enzyme-substrate adduct-complex interchanging. Now, with Equation (18) back in general conservation probability Equation (15) and comparing the result with the basic Michaelis-Menten equation (1) there is immediate to derive the associated unreacted probability expression:

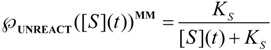
(19)

At this points one remarks Equation (19) fulfilling both limiting conditions of Equation (17); it may be eventually replaced with a more general formulation:

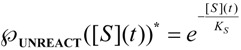
(20)
that jointly satisfies the same set of constraints. The equivalence between eqs. (19) and (20) is achieved by performing the 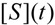 first order expansion for the latter case where the bound substrate approaches zero limits, *i.e.* within the low substrate concentrations.

**Figure 1:**
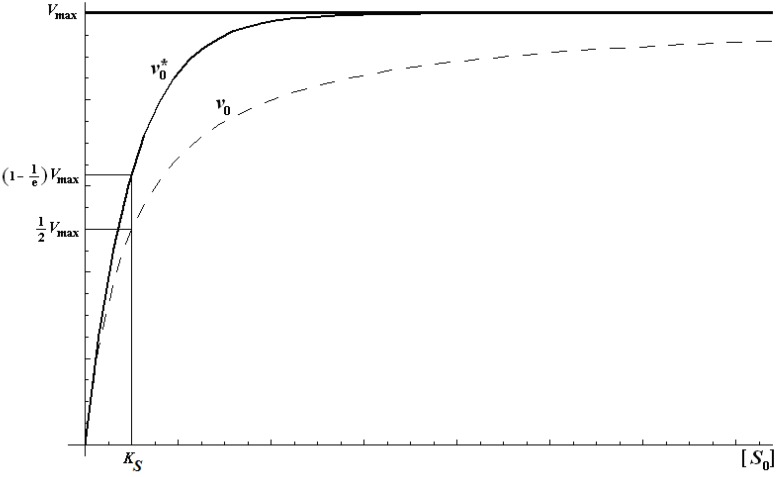
Michaelis-Menten and logistic initial velocities plotted against initial substrate concentration for the E-S mono-substrate enzymic reaction. The dashed curve corresponds to the Michaelis-Menten equation (1) while the continuous thick curve represents its logistic generalization from (21): 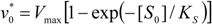.

However, by using equation (20) instead of (19) the actual kinetic equation has the exponential form:

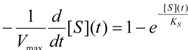
(21)
with a behavior encompassing the same kinetic parameters as the original Michaelis-Menten equation (1), yet displaying faster substrate consumption, see Figure 1.

Notably, the modified Michaelis-Menten equation (1) under the exponential form (21) has also the additional advantage in providing *elementary analytical* solution for the substrate progress curve. This can be immediately obtained by integrating Equation (21):

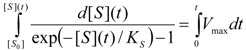
(22)
leaving with the new equation to be solved:

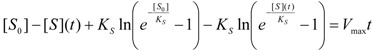
(23)
which is only apparently transcendental. More practically, by performing the working substitution:

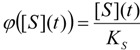
(24)
one rewrites Equation (23) under the simple form:

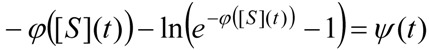
(25)
where we have also introduced the function notation:

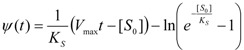
(26)

Now, the exact solution of equation (25) takes the logarithmic form:

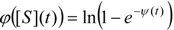
(27)
allowing, through replacing the notations (24) and (26), to call the obtained solution as the *logistic progress curve* expression:

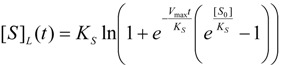
(28)

In fact, the form (28) successfully substitutes by an elementary logarithmic dependency the W-Lambert implicit solution (4) [31,32,33,34]. Moreover, through comparing the Michaelis-Menten temporal substrate solutions under eqs. (4) and (28), the so called *logistic transformation* may be formulated [32]:

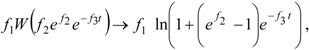
(29)

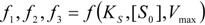
(30)
this way allowing the direct formulation of the analytic progress curves once the W-Lambert counterpart form is known. This probabilistic framework and its results will be in foregoing section applied to the present Haldane-Radić equation (14).

## 3. Results and Discussion

### 3.1. Probabilistic form of the Haldane-Radić Equation

The probability form (15) of Equation (14) may be immediately inferred by considering the reactive term as in (18) and rewriting the Haldane-Radić equation as:

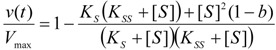
(31)

From Equation (31) the unreactive term may be recognized as being composed by two parts: the Michaelis-Menten contribution (19) superimposed on the specific Haldane-Radić term, namely:

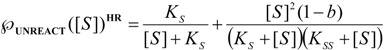
(32)

Interestingly, when performing the limits prescribed by general conditions (17) one gets:

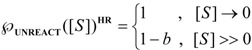
(33)
while noting the persistent non-zero non-reactive behavior for higher substrate concentration – a feature that accounts for the inhibition character calling the Haldane specificity. However, the general conditions (17) are fully recovered by sending the *b* parameter to 1, which corresponds from (32) with resembling the Michaelis-Menten unreactive term (19):

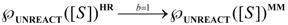
(34)

Up to now, the presented probabilistic analysis shows it is qualitatively compatible with the general Haldane-Radić to Michaelis-Menten conditions (7); the quantitative issue will be in the sequel addressed.

### 3.2. Temporal Solution of Haldane-Radić Equation by W-Lambert Functional

The starting kinetic equation under the form (14) is firstly rewritten as:

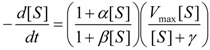
(35)
with the introduced notations:

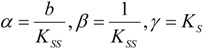
(36)
It may be further rearranged as the ordinary differential equation:

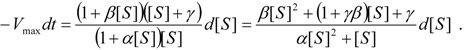
(37)
Next, Equation (37) may be transformed through performing the polynomial ratio with the result:

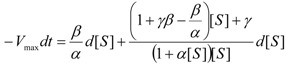
(38)
which is formally ready for integration.

However, another notation will simplify the analytical discourse, namely:

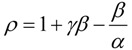
(39)
with the help of which the integration of Equation (38), between the initial conditions 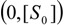 an the current one 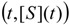, it firstly yields:

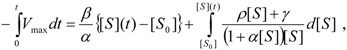
(40)
while, by means of the right hand last term decomposition:

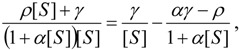
(41)
it leaves with the result:

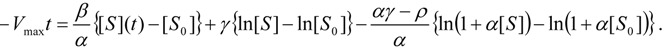
(42)

Now, equation (42) may be seen under the form:

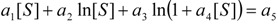
(43)
that may be simplified to the compact expression:

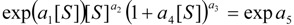
(44)
with the shortcuts:

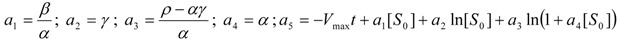
(45)

Worth noting that equation (44) looks as a modified form of the Euler-Lambert equation (5). Yet, the polynomial appearance in (44), viz.:

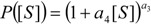
(46)
produces a modification in the Euler-Lambert equation (5) that now becomes:

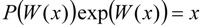
(47)
with essentially non-algebraic analytic solutions so far. Instead, if we consider a generalization form of the polynomial (46) such that:

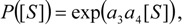
(48)
one recovers the W-type equation:

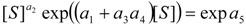
(49)
that may be solved to give:

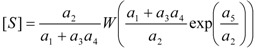
(50)

Note that the equivalence between eqs. (46) and (48) relays on the limiting case:

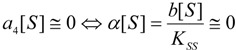
(51)
being such condition adding also the low substrate environment to those characterizing the Haldane-Radić to Michaelis-Menten reduction scheme, see Equation (7). However, the question whether condition (51) suggests the low substrate constraint as sufficient or alternatively to the classical conditions (7) for that the Haldane-Radić to Michaelis-Menten reduction may be achieved is to be further explored.

Going back to the notations in (45) the W-Lambert form of the substrate depletion (50) looks like:

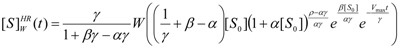
(52)
or in original kinetic parameters, throughout the shortcuts (36) and (39), casts as:

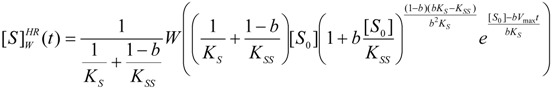
(53)

Further use of this progress curve is in next discussed.

### 3.3. Temporal Solution of Haldane-Radić Equation by Analytic Logistic Transformation

Despite the formal solution (53) was achieved, it still suffers from a lack in analytical shape since the W-Lambert poses an implicit functional character. In order to improve such implicit solution one can consider in (52) the same binomial to exponential transformation for initial substrate [S_0_] as previously performed for the instantaneous free substrate [S](t), see eqs. (46) and (48), viz.:

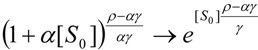
(54)

Under these conditions, Equation (52) becomes:

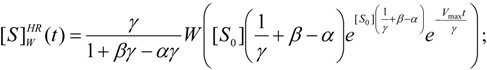
(55)

Equation (55) may be turned into an analytical expression once the *logistic transformation* is employed according with the general recipe of Equation (29) to provide the logistic substrate temporal form:

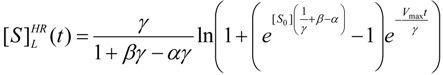
(56)
or, with replacement of the kinetic parameters from (36) and (39), the progress curve:

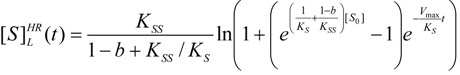
(57)

Worth noting that the logistic solution (57) finely tunes the extreme conditions for the substrate kinetics, namely:

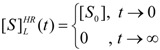
(58)
a matter not easy to verify using the W-Lambert expressions (4) or (55).

From now on, with the help of expression (57) the temporal course of the kinetics (14) may be formulated in an analytical manner by employing the required temporal derivative of the substrate:

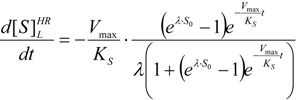
(59)
with the working parameter:

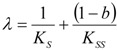
(60)

Thus, the initial velocity for product formation, *i.e.*, at *t* = 0, is:

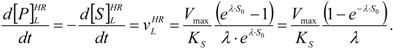
(61)

**Figure 2:**
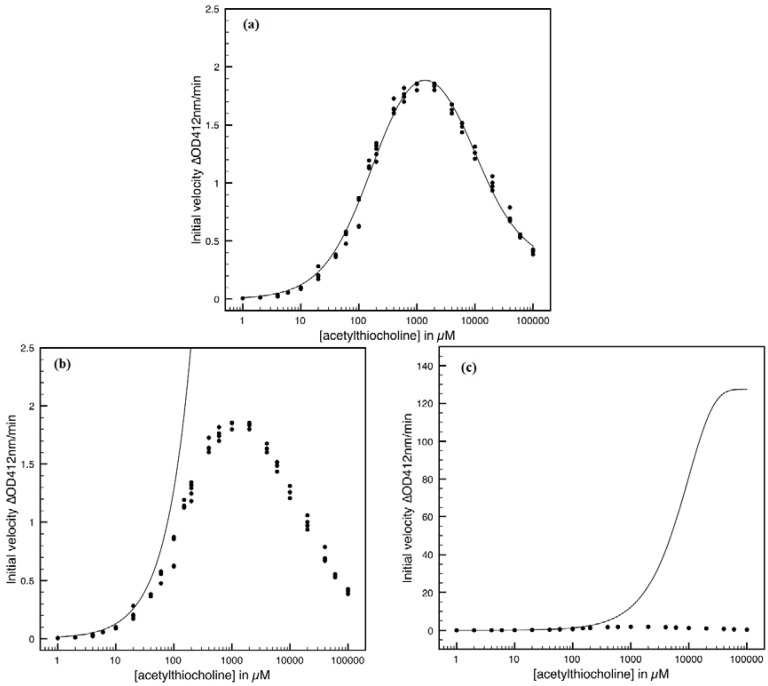
(a): The fitting curves for original Haldane-Radić velocity equation (14) corresponding with the Scheme 2 for large human acetylthocholine substrates’ concentration intervals; (b) & (c) the same fits with logistic based velocity equation (61) corresponding to the “reduction” of the Scheme 2 to the consecrated Michaelis-Menten mechanism of Scheme 1; the kinetic fitting parameters are *K_S _*= 190 ± 30 µM; *K_SS_* = 8,700 ± 2,200 µM ; *V_max_* = 2.45 ± 0.15 ∆OD(optical density)/min; *b* = 0.12 ± 0.03.

This curve may be used to perform the non-temporal fit for kinetic parameters, with the caution however that it gives best results in the low initial substrate range, according with the equivalence (51). For instance, for human AChE, using acetylthiocholine as the substrate, one gets the full bell-shaped curve fit of Figure 2(a) by using Haldane-Radić equation (14) for the working parameters *K_S _*= 190 ± 30 µM; *K_SS_* = 8,700 ± 2,200 µM ; *V_max_* = 2.45 ± 0.15 ∆OD/min; *b* = 0.12 ± 0.03; instead, with the same parameters in logistic related derived velocity of Equation (61) the departure is recorded for initial substrate concentrations higher than 100 µM, see Figure 2 (b), targeting the Michaelis-Menten kinetics of Figure 1 in Figure 2(c).

**Figure 3:**
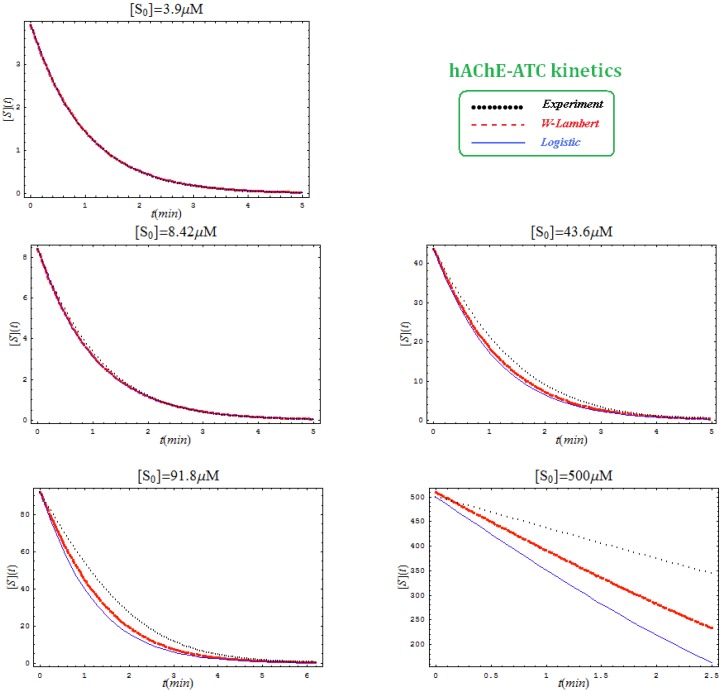
The W-Lambert and logistic progress curves as they fit with experimental data for the hAChE-ATC kinetics, according to the equations (55) and (57), through considering the kinetic parameters from (36) and (39) with the actual values *K_S _*= 160 µM; *K_SS_* = 8,700 µM; *V_max_* = 162.45 µM/min; *b* = 0.12, for various initial substrate concentrations.

**Figure 4:**
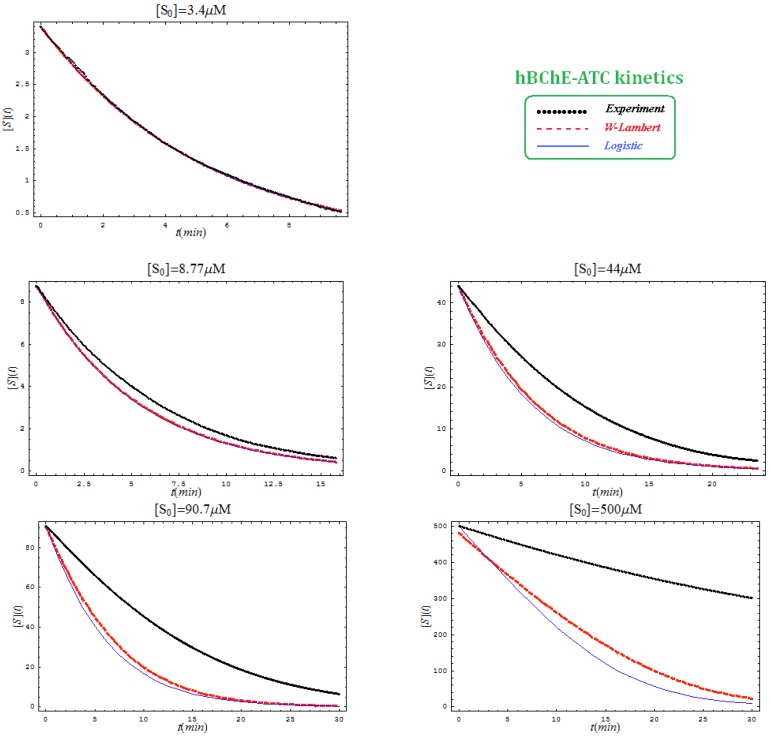
The same type of plots as in Figure 3, here for hBChE-ATC kinetics and parameters *K_S _*= 160 µM; *K_SS_* = 8,700 µM; *V_max_* = 31.0 µM/min; *b* = 3.

**Figure 5:**
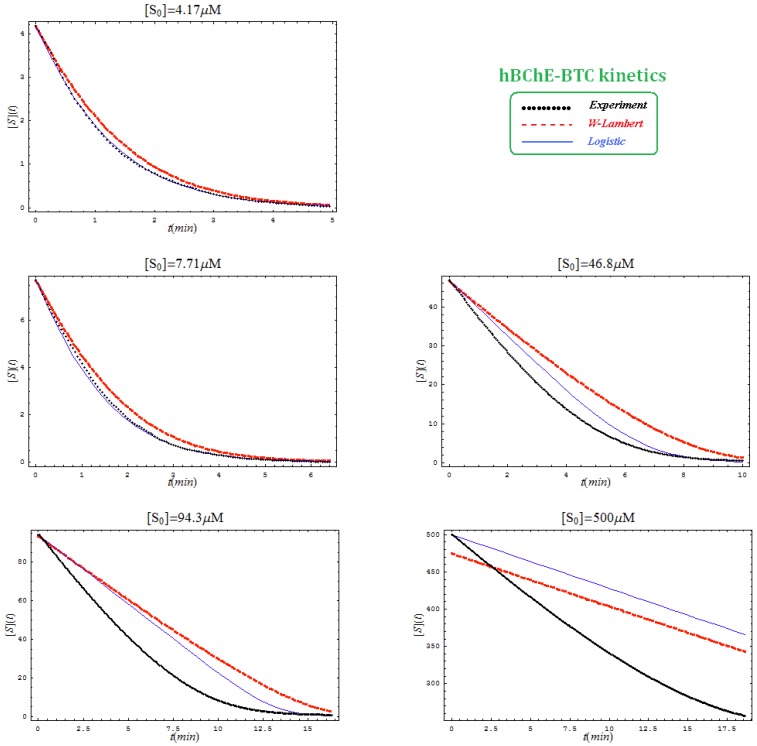
The same type of plots as in Figure 3, here for hBChE-BTC kinetics and parameters *K_S _*= 7.5 µM; *K_SS_* =8,700 µM; *V_max_* = 7.2 µM/min; *b* = 3.

**Figure 6:**
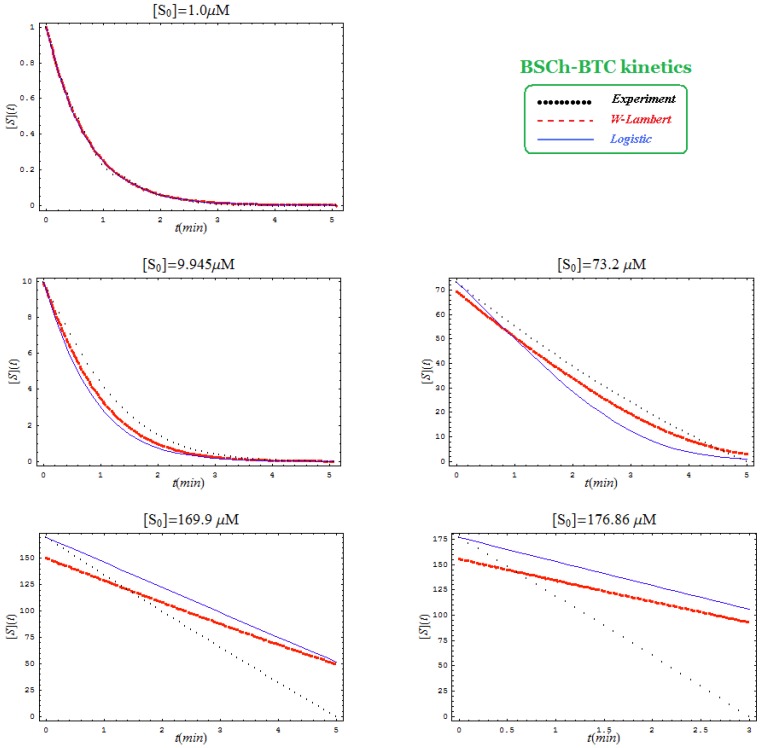
The W-Lambert and logistic progress curves as their fit with the experimental data for hydrolysis of various concentrations of butyrylthiocholine by a fixed concentration of butyrylcholinesterase according to the equations (55) and (57), through considering the kinetic parameters from (36) and (39) with the actual values *K_S _*= 7.5 µM; *K_SS_* = 8,700 µM; *V_max_* = 7.2 µM/min; *b* = 3, for various initial substrate concentrations.

This is not surprisingly, since the actual Haldane-Radić W-Lambert and logistic progress curves were obtained through modification of the analytic conditions of the Scheme 2 such that being “reduced” or “absorbed” to the Scheme 1 for the lower concentration of the substrate. To check the consistency of this hypothesis also for the progress curves the Figure 3, Figure 4, Figure 5 and Figure 6 display the fitting of the above W-Lambert and logistic equations (55) and (57) for various experimental enzymic kinetics with the fitting parameters of equations (36) and (39) determined for the lower substrate concentration and then tested for higher and higher values of it.

The analysis of the plots of Figure 3, Figure 4, Figure 5 and Figure 6 illustrates the interesting recorded behavior:

- The hAChE-ATC kinetics (Figure 3) differs from hBChE-ATC kinetics (Figure 4) essentially only in the lowering the *V_max_* and increasing *b* parameters for the last case, in accordance with the prescription associated with activation mechanism; moreover, the W-Lambert and logistic curves depart clock-wise from experimental record and more quickly for logistic case;
- The hBChE-ATC kinetics (Figure 4) differs from hBChE-BTC kinetics (Figure 5) essentially by further lowering the *V_max_* accompanied by decrease of *K_S_* parameter for the BTC kinetics, while the W-Lambert and logistic computationally fitting curves show in Figure 5 a departure tendency in anti-clock-wise respecting the experimental evidence; here is also recorded the clear failure of the numerical W-Lambert progress curve to reach the initial substrate concentration, a matter fully satisfied by the logistic counterpart instead;
- Comparison between hBChE-BTC kinetics (Figure 5) and BSCh-BTC hydrolysis (Figure 6) reveals that by maintaining the same kinetic parameters between these two cases, in the latter, the computational fitting with respect the experimental data oscillate from clock-wise to anti-clock-wise departure of the logistic model as the initial substrate concentration goes from lower (<100 µM) to higher (>100 µM) values, respectively; here, again, the initial time discrepancy between W-Lambert and logistic kinetics is obviously in the favor of the latter approach.

However, the present analysis allows the general rules:

- The Haldane-Radić kinetics may be quite well modeled by its Michaelis-Menten counterpart progress curves for substrate kinetics below 100 µM in all studied cases, being this condition susceptible to be a general fact that is independent of ideal approach for the Haldane-Radić kinetic parameters *K_SS_* and *b* as prescribed in Equation (7);
- Haldane-Radić kinetics display full specificity in looping S-E mechanisms of inhibition/activation for higher concentration of the substrate, *i.e.* within the mili-molar range;
- The W-Lambert logistic fails to behave correctly at initial time of kinetics in the case of higher initial substrate concentrations (see Figure 3, Figure 4, Figure 5 and Figure 6); however, the logistic counterparts always correct this flaw due the analytical limit (58).

## 4. Conclusions

Although with great impact in modeling the synergism between the active site gorge and peripheral sites of wild and mutant enzymes, the complex Haldane-Radić kinetics mechanism may be reduced to the more tractable Michaelis-Menten mechanism under special conditions of substrate-enzyme interactions. Actually, through performing a detailed analytical analysis of the initial velocity and progress curves, at both conceptual-analytical and computational-numerical levels for various cholinesterases systems, there follows that such reduction is possible in either of the kinetic conditions:

- Higher dissociation constant for inhibition/activation substrate site interaction to enzyme;
- Equal catalytic efficiency of inhibition/activation substrate-enzyme loop as provided by E-S hydrolysis;
- Lower substrate concentration, typically in the range up to the 100 µM.

It was proved that all these three conditions may be regarded as equivalent in reducing Haldane-Radić to Michaelis-Menten enzymic kinetics, yet, being the last one a new one added, specific to some mutant *in vivo* enzyme kinetics of cholinesterases, though the present detailed theoretical and fitting analysis show its sufficiency the envisaged reduction taking place even when the first two conditions do not apply.
